# Frozen Mother’s Own Milk Can Be Used Effectively to Personalize Donor Human Milk

**DOI:** 10.3389/fmicb.2021.656889

**Published:** 2021-04-14

**Authors:** Monica F. Torrez Lamberti, Natalie A. Harrison, Marion M. Bendixen, Evon M. DeBose-Scarlett, Sharon C. Thompson, Josef Neu, Leslie Ann Parker, Graciela L. Lorca

**Affiliations:** ^1^Department of Microbiology and Cell Science, Genetics Institute, Institute of Food and Agricultural Sciences, University of Florida, Gainesville, FL, United States; ^2^College of Nursing, University of Florida, Gainesville, FL, United States; ^3^Division of Neonatology, Department of Pediatrics, College of Medicine, University of Florida, Gainesville, FL, United States

**Keywords:** mother’s own milk, donor human milk, frozen milk, microbiota, microbial expansion, restored microbiota human milk

## Abstract

Feeding preterm infants mother’s own milk (MOM) lowers rates of sepsis, decreases necrotizing enterocolitis, and shortens hospital stay. In the absence of freshly expressed MOM, frozen MOM (FMOM) is provided. When MOM is unavailable, preterm infants are often fed pasteurized donor human milk (DHM), rendering it devoid of beneficial bacteria. We have previously reported that when MOM is inoculated into DHM to restore the live microbiota [restored milk (RM)], a similar microbial diversity to MOM can be achieved. Yet, it is unknown if a similar diversity to MOM can be obtained when FMOM is inoculated into DHM. The goal of this study was to determine whether a similar microbial composition to MOM could be obtained when FMOM is used to personalize DHM. To this end, a fresh sample of MOM was obtained and divided into fresh and frozen fractions. MOM and FMOM were inoculated into DHM at different dilutions: MOM/FMOM 10% (RM/FRM10) and MOM/FMOM 30% (RM/FRM30) and incubated at 37°C. At different timepoints, culture-dependent and culture-independent techniques were performed. Similar microbiota expansion and alpha diversity were observed in MOM, RM10, and RM30 whether fresh or frozen milk was used as the inoculum. To evaluate if microbial expansion would result in an abnormal activation on the innate immune system, Caco-2 epithelial cells were exposed to RM/FRM to compare interleukin 8 levels with Caco-2 cells exposed to MOM or DHM. It was found that RM samples did not elicit a significant increase in IL-8 levels when compared to MOM or FMOM. These results suggest that FMOM can be used to inoculate DHM if fresh MOM is unavailable or limited in supply, allowing both fresh MOM and FMOM to be viable options in a microbial restoration strategy.

## Introduction

The American Academy of Pediatrics recommends all preterm infants receive exclusive mother’s own milk (MOM) or donor human milk (DHM) when MOM (fresh or frozen) is not available (Eidelman and Schanler, 2012). Early life may represent a critical window of gut bacteria acquisition that affects intestinal bacterial composition and immune system development in infancy through adulthood (Nash et al., 2017). Important for the preterm infant, MOM reduces the incidence and severity of infections, especially necrotizing enterocolitis and late-onset sepsis (Schanler et al., 2005; Goulet, 2015; Jost et al., 2015; Cortez et al., 2018). MOM feedings impact both innate and acquired immunity, aids in digestion, and decreases cost of neonatal intensive care unit (NICU) care (Eidelman and Schanler, 2012; Fernández et al., 2013; Meier and Bode, 2013; Moles et al., 2015). Additionally, mothers may benefit from the protective effects against breast, ovarian cancer, and diabetes associated with lactation (Belfort et al., 2016).

Mothers of infants requiring admission to a NICU must express their milk via a breast pump (pump dependent) to establish and maintain their milk supply due to mother/infant separation and the infant being too immature or ill for direct feeding at the breast. It is well known that pump-dependent mothers often produce insufficient amounts of MOM to provide exclusive MOM feedings (Eidelman and Schanler, 2012). As a result, DHM is used when MOM is unavailable. DHM is obtained from pooled donors, pasteurized and frozen (HMBANA Guideline Committee, 2020). This process renders the milk devoid of live commensal bacteria. MOM is a unique and complex mixture that not only contains nutritional components and immune modulators, but is also a rich and personalized microbiota (Chatterton et al., 2013; Andreas et al., 2015; Demmelmair and Koletzko, 2017; Ruiz et al., 2017; Dror and Allen, 2018). Several studies describe the human milk microbiota as a dynamic community represented mostly by members of the Proteobacteria, Firmicutes, and Actinobacteria phyla (Hunt et al., 2011; Cacho et al., 2017; Togo et al., 2019). The variability observed in the composition of the milk microbiota may be attributed to different variables, such as diet, race, human milk oligosaccharides secretor status, mode of delivery, the mother’s skin, and the infant’s mouth (Novak and Innis, 2011; Cabrera-Rubio et al., 2012, 2019; Biagi et al., 2017; Cacho et al., 2017; Vaidya et al., 2017; Xi et al., 2017).

Milk microbiota represents the infant’s first experience with microbes; this contact is important to the development of the immature immune system, and it may contribute to building a recognition network of commensal non-pathogenic bacteria. In our previous study, we tested the hypothesis that DHM microbiota could be restored using MOM as the inoculum. A similar microbial diversity to the individual MOM can be reached in the microbiota restored milk (RM) after 4 h of incubation at 37°C using freshly expressed MOM (Cacho et al., 2017). Using culture-independent techniques, we found that the most abundant genera in MOM were *Halomonas*, *Shewanella*, *Corynebacterium*, *Staphylococcus*, and *Lactobacillus*, whereas *Acinetobacter*, unclassified Enterobacteriaceae, and *Serratia* were most common in pasteurized DHM (Cacho et al., 2017). A comparative metabolomic analysis of DHM, MOM, and RM samples showed that more than 1,000 features were significantly different between MOM and DHM milk. Moreover, significant changes were observed in MOM and RM30 over time, whereas the concentration of metabolites in DHM remained the same, denoting that these variations in the metabolites are linked to the microbial expansion (Torrez Lamberti et al., 2020).

The provision of recently expressed MOM fed to preterm infants is prioritized in current practice, followed by frozen MOM (FMOM), and then DHM when MOM is not available in sufficient quantities. Yet, it is unknown whether the freezing step in FMOM may alter the viability and survival of the microbiota and whether or not it can be used to personalize DHM following the same strategy developed for freshly expressed MOM. This research seeks to expand on the personalization of DHM to include FMOM and optimize a shorter incubation time to resemble clinical practice more closely in the NICU. Our aim was to evaluate whether FMOM and fresh MOM can equally be used to inoculate pasteurized DHM to obtain enriched commensal microbes and their beneficial metabolic interactions similar to MOM. We hypothesize that the inoculation of DHM with FMOM will produce a personalized milk containing the unique mother’s microbiota comparable to fresh MOM.

## Materials and Methods

### Study Subjects and Setting

The study setting was a 72-bed unit level IV regional NICU in the Southeastern United States drawing from semirural and rural catchment areas. The prospective quasi-experiment pilot study was approved through the University of Florida Institutional Review Board (IRB201400527). Between January 2019 and June 2019, a convenience sample of 11 mothers along with her infant, delivered less than 34 weeks’ gestational age and admitted to the NICU, was recruited. Mothers were included if they were expressing MOM of more than 100 mL/d, producing at least 45 mL MOM at an expression session, 18 years of age, and spoke English. A mother was excluded if she had taken an antibiotic within the past 3 days, delivered an infant who was severely ill, or had a chromosomal abnormality. Written informed consent was obtained from each recruited mother–infant pair.

### Sample Collection

Each mother provided a single fresh MOM sample collected utilizing a sterile Symphony^®^ double breast pump kit at a single expression session with an electric hospital Symphony^®^ breast pump (Medela, McHenry, IL). The fresh MOM sample was at least 45 mL, which was obtained from both breasts. Beyond standard handwashing and expression per NICU protocol, no breast hygiene preparation was performed to mimic the infant exposure to the mother’s breast microbiota. To minimize the risk of contamination, the same study staff member assisted the mother with the expression session and sample collection. Pasteurized DHM from the same frozen batch was procured from the Human Milk Banking Association of North America (HMBANA). One fecal sample from the infant of each mother was collected directly from the diaper. Samples were transported immediately on ice to the nearby microbiology laboratory and then stored at −80°C for later DNA extraction and 16S rRNA gene sequencing.

### Mediterranean Diet Adherence Screener

During the single expression session, the Mediterranean Diet Adherence Screener (MEDAS) questionnaire was administered verbally. The MEDAS instrument determines the level of dietary adherence to a Mediterranean diet (MD) eating pattern (Martínez-González et al., 2012). A value of 1 is attributed to each multiple-choice food frequency question. Three categories of MD adherence are determined by low (0–5), average (6–9), and greater than or equal to 10 for high adherence. The 14-item MEDAS questionnaire has been adapted to the English language and validated in the southeastern United States with a population comparable to a large public university urban center with surrounding rural areas (Bottcher et al., 2017). MEDAS was validated in pregnant women delivering prematurely noting the question regarding wine consumption was eliminated (Papazian et al., 2019; Parlapani et al., 2019). Yet, the level of MD adherence from these studies shows a different measured category from the MEDAS trial (a single cutoff point as ≤ 7 for low or high MD adherence). In limited studies using MD adherence during pregnancy/postpartum, Papazian et al. found the tools measuring MD adherence were varied and heterogeneous and for which different cutoff points were applied (Papazian et al., 2019). As no consensus has been established for the pregnant/postpartum mother, we chose to keep the wine question and maintain the scoring categories of the original MEDAS trial.

### Microbial Restoration Strategy

The microbial restoration strategy was as previously described in Cacho et al. (2017) with the following modifications (Supplementary Figure 1). Each fresh MOM sample was divided into two fractions: fresh MOM (MOM and RM) or frozen for 24 h (FMOM and FRM). The fresh MOM fraction was processed immediately, whereas the FMOM fraction was processed after 24 h at −20°C. Samples were inoculated in DHM at dilutions of 10 and 30% (vol/vol) and incubated at 37°C for 4 h. Samples were taken at three time points: time 0 (T0), 2 h (T2), and 4 h (T4), and analyzed using culture-dependent and -independent techniques.

### Culture-Dependent Bacterial Analysis

At each time point, total viable cell counts were performed. Serial dilutions of each sample were plated on selective and non-selective agar plates at time 0, 2, and 4 h (T0, T2, and T4, respectively). Based on our previous study and the most common groups of bacteria isolated from human milk in other studies (Wight, 2001; Martín et al., 2003; Reviriego et al., 2005; Jost et al., 2013; Menon and Williams, 2013; Cacho et al., 2017), the following growth media were used: acidified Man, Rogosa, and Sharp (MRS) agar (pH 5.5) for lactic acid bacteria (peptone 10 g, meat powder 10 g, yeast peptone 5 g, table sugar 20 g, K_2_HPO_4_ 2 g, sodium acetate 5 g, ammonium citrate tribasic 2 g, MgSO_4_⋅7H_2_O 0.2 g, MnSO_4_⋅H_2_O 0.05 g, tween 80 1 g, agar 15 g; final volume of 1 L with DI water), mannitol salt agar for *Staphylococcus* (Hardy Diagnostics Santa Maria, CA, United States), nutrient broth agar for facultative aerobes including *Streptococcus* (Research Products International, Mt. Prospect, IL, United States), 5% sheep blood trypticase soy agar (TSA agar) for fastidious organisms and for the visualization of hemolytic reactions (Hardy Diagnostics Santa Maria, CA, United States), and MacConkey agar for enterobacteria (Hardy Diagnostics Santa Maria, CA, United States). All plates were incubated at 37°C for 48–72 h. MRS agar plates were incubated in jars enclosed with a burning candle to create a reduced oxygen environment, whereas 5% sheep blood TSA, mannitol agar, MacConkey, and nutrient agar plates were incubated aerobically.

### DNA Isolation, Library Construction, and Sequencing

DNA was extracted from milk samples and preserved at −80°C using a classic phenol–chloroform extraction as described in Quigley et al. (2012) and Volk et al. (2014). DNA from stool samples was isolated using QIAamp^®^ PowerFecal^®^ Pro DNA kit (QIAGEN). The following modification was applied to both protocol extraction: 100 μL of protease from *Streptomyces griseus* 20 mg/mL (Sigma–Aldrich, Steinheim, Germany) was added (Marcial et al., 2017). The mixture was incubated at 37°C for 15 min, and then, the samples were processed according to the protocol. In the elution step, the DNA was collected in 50 μL of water and quantified. The DNA concentration was standardized to 1 ng/mL before amplification of the V4 region using primers 515F/806R barcoded for Illumina HiSeq platform (Caporaso et al., 2012). To reduce variability and potential bias from potential sources of DNA contamination, all samples were processed with the same batch of DNA extraction kits as well as polymerase chain reaction reagents.

### Bioinformatics, Normalization, and Statistical Analysis

Clustering of operational taxonomic units (OTUs) at 97% similarity was performed with the subsampled open-reference OTU picking method (Rideout et al., 2014) with no removal of singletons. The Silva reference dataset version 138 (Quast et al., 2013) was used as the reference for OTU picking and for taxonomy assignment using DADA2 R package v1.10 (Callahan et al., 2016). OTUs identified as mitochondrial DNA were removed from further analyses using R studio. Community structure was analyzed in R with phyloseq, Vegan, and Microbiome packages (Philip, 2003; Holmes, 2013). Cumulative sum scaling was used to normalize the counts. The plots were generated with ggplot2 (Wickham, 2009). Differences in taxonomic profiles were analyzed by Wilcoxon rank sum test (for two groups) or by Kruskal–Wallis rank sum test (for multiple groups). Permutational multivariate analysis of variance (PERMANOVA) was used to analyze clustering significance. To investigate MOM/FMOM contribution, each MOM/RM10/RM30/DHM (fresh or frozen) set was normalized to the counts found in the corresponding DHM at T0 and adjusted to the dilution rate of MOM (10 or 30%).

### Interleukin 8 Caco-2 Cell Stimulation Assay

Human intestinal Caco-2 cells were cultured at 37°C in Dulbecco modified eagle culture medium (DMEM) supplemented with 15% fetal bovine serum (Invitrogen), 2% of 10,000 units of penicillin, 10 mg streptomycin, and 25 μg amphotericin B per mL (Sigma-Aldrich) in a humidified atmosphere (5% CO_2_). For assays, the number of cells per well was estimated by counting the cells in a Neubauer hematocytometer chamber and seeded in six-well plates (1 × 10^5^ cells/well). The cells were treated with 4% of defatted milk (vol/vol) in DMEM supernatants for 24 h. The culture supernatants were tested by enzyme-linked immunosorbent assay (ELISA) for production of interleukin 8 (IL-8) (BD OptEIA^TM^). Basal level of IL-8 in the milk samples was quantified and subtracted from the Caco-2 cell supernatants. The experiment was performed with biological and technical triplicates. Data were analyzed using analysis of variance (ANOVA), and Tukey honestly significant difference method was used to assign statistical significance for a *p* < 0.05.

## Results

### Demographics and MEDAS Adherence

A cohort of 11 mothers, 45.5% Caucasian, 45.5% African American, and 1% Asian, with a mean age of 29 ± 5.5 years, was included in this study (Table 1). To determine the diet homogeneity among mothers, a MEDAS questionnaire was provided (Bottcher et al., 2017). Most mothers (82%, *n* = 9) showed low adherence to an MD, and 18% (*n* = 2) showed medium adherence (Table 1). A high percentage, 73%, of the infants included were males (*n* = 8), and females accounted for the remaining 27% (*n* = 3), whereas 64% (*n* = 7) of infants were born by cesarean section and 36% (*n* = 4) by vaginal delivery (Table 1).

**TABLE 1:** Demographics of mothers and infants.

**Infant demographics**	** (*N* = 11)**
Gestational age at birth (weeks)	30 ± 2
Birth weight (grams)	1481 ± 512.7
**Gender**	
Male	73% (*n* = 8)
Female	27% (*n* = 3)

**Maternal demographics**	**(*n* = 11)**

Maternal age (years)	29 ± 5.5
Maternal BMI	33 ± 6.3
**Delivery mode**	
Vaginal	36% (*n* = 4)
Cesarean section	64% (*n* = 7)
Planned cesarean section	43% (*n* = 3)
**MEDAS questionnaire**	
Low	82% (*n* = 9)
Medium	18% (*n* = 2)
High	0% (*n* = 0)

**Maternal/infant**	**(*n* = 11)**

**Race**	
Caucasian	45.5% (*n* = 5)
African American	45.5% (*n* = 5)
Asian	9% (*n* = 1)

### Donor Human Milk Can Be Personalized With FMOM

Previously, it was found that 30% of fresh MOM inoculated in DHM (RM30) and incubated for 4 h resulted in microbial expansion (Cacho et al., 2017). Because recently expressed MOM is not always available, here we investigate whether the freezing step in FMOM would affect the ability of the microbiota to personalize DHM. To this end, the microbiota resaturation process was performed using both MOM and FMOM to inoculate DHM at 10% and 30% dilution rates followed by 4 h of incubation. Culture-dependent and culture-independent methods were used to analyze the milk microbiota restoration process. For the culture-dependent method, different bacterial culture media were utilized to quantify fluctuations in the bacterial populations over time. It was found that DHM did not result in colony-forming units (CFU/mL) in any of the media tested, whereas FMOM microbiota can efficiently expand after having been stored at −20°C. Similar CFU/mL were observed at T0 of incubation between MOM and FMOM, reaching similar levels of bacterial expansion after 4 h of incubation.

**FIGURE 1:**
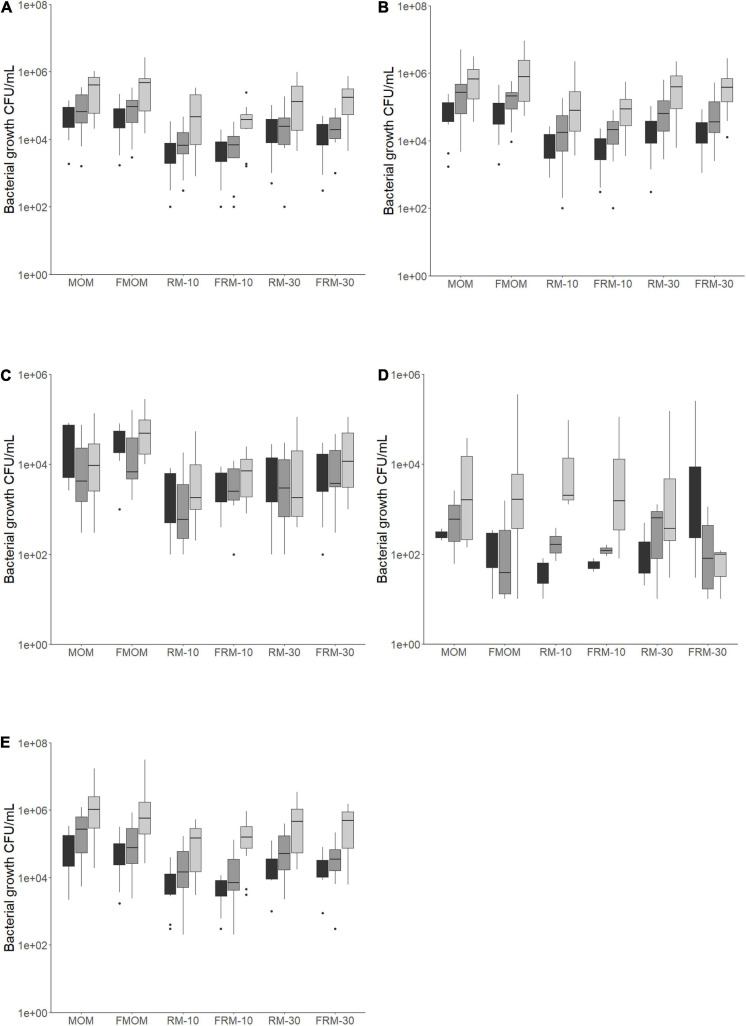
Bacteria load for each milk and personalized DHM samples (MOM, FMOM, RM10, FRM10, RM30, and FRM30) in different types of media at time = 0 (T0, black boxes) and incubated over 2 h (T2, gray boxes) and 4 h (T4, light gray boxes) at 37°C. **(A)** CFU/mL in nutrient agar media; **(B)** CFU/mL in mannitol salt agar media; **(C)** CFU/mL in MRS agar media; **(D)** CFU/mL in MacConkey agar media; **(E)** TSA 5% blood agar media. FMOM, frozen MOM samples; FRM10, frozen RM10 samples; FRM30, frozen RM30 samples; MOM, fresh MOM samples; RM10, fresh RM10 samples; RM30, fresh RM30 samples.

In nutrient and mannitol salt agar plates, the load of bacteria was between 10^3^ and 10^5^ CFU/mL, with most of them being greater than 10^4^ CFU/mL in both MOM and FMOM (Figures 1A,B). In contrast, only 40% of the MOM samples grew on MacConkey agar plates at concentrations between 10^1^ and 10^5^ CFU/mL (Figure 1D), showing no significant differences between MOM and FMOM.

The bacterial load in MRS media, which targets lactic acid bacteria, was between 10^3^ and 10^5^ CFU/mL in both MOM and FMOM samples (Figure 1C) in agreement with our previous report (Cacho et al., 2017). Interestingly, while a decrease in the load of bacteria was observed after 4 h of incubation of MOM/RM samples in MRS agar, an increase was observed in FMOM/FRM samples over time (Figure 1C); however, these differences were statistically significant only when MOM and FMOM samples were compared at T4 of incubation (*p* < 0.05) (Figure 1C).

In 5% sheep blood TSA agar, no significant differences between MOM/RM and FMOM/FRM were observed with viable counts ranging from 10^3^ to 10^7^ CFU/mL (Figure 1E). However, as this media allows the discrimination between hemolytic activity, significantly lower numbers (*p* < 0.05) of γ-hemolytic bacteria (non-hemolytic) were observed in FMOM/FRM samples when compared to its fresh counter parts at T4 (Supplementary Figure 2A). Differences, although not significant, were observed as well as in α- and β-hemolytic bacteria counts between MOM/RM and FMOM/FRM samples (Supplementary Figures 2B,C).

In summary, it was shown that microbial expansion was equally effective using MOM or FMOM; however, the inoculation of DHM with 10% or 30% of FMOM resulted in the differential expansion of γ-hemolytic bacteria, as well as higher CFU/mL of lactic acid bacteria.

### The Use of FMOM in Microbial Restoration Has No Impact in the Alpha Diversity

**FIGURE 2:**
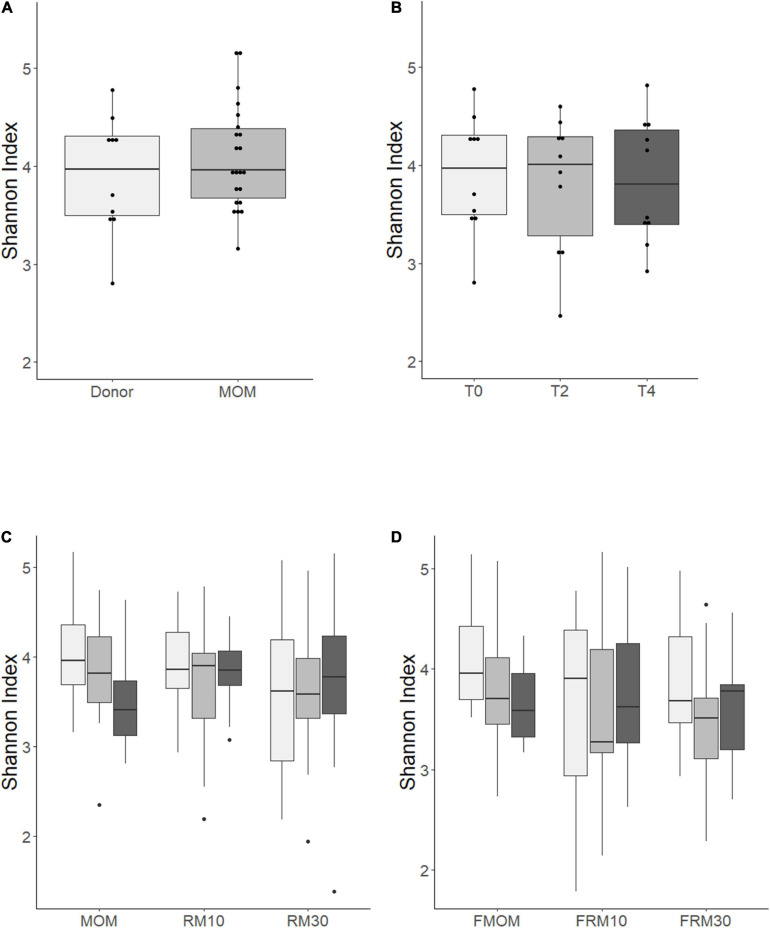
Alpha diversity determination over time using Shannon index of: **(A)** MOM (gray box) versus DHM (light gray box) at T0; **(B)** DHM samples, **(C)** MOM and RM samples, **(D)** FMOM and FRM samples at time 0 (T0, light gray boxes) and incubated over 2 h (T2, gray boxes) and 4 h (T4, black boxes) at 37°C. Statistical significance was determined using ANOVA followed by Kruskal–Wallis tests.

To investigate if the freezing process had an impact on the diversity of microbiota that was expanded in the RM samples, Illumina sequencing of the V4 region of the bacterial 16S rRNA was performed on all milk samples. The alpha diversity expressed as Shannon index showed no significant differences between MOM and DHM samples at T0 (Figure 2A) or DHM samples over time (Figure 2B), in agreement with our previous report (Cacho et al., 2017). When the diversity was evaluated over time, trends toward a decrease in microbiota diversity were observed in MOM samples (*p* = 0.08) and FMOM (*p* = 0.10), but not in RM/FRM groups (Figures 2C,D). Clustering was observed when the communities were compared using non-metric multidimensional scaling (NMDS) (*R*^2^ = 0.02587 *p* < 0.001) and principal coordinates analysis (PCoA) (*R*^2^ = 0.0255 *p* < 0.001) regarding the personalization process (Supplementary Figures 3A,B).

To evaluate the contribution of MOM or FMOM microbiome expansion in the RM samples, the DHM background was removed from each RM10 and RM30 set of samples and normalized to the dilution rate of MOM (10 or 30%). Multidimensional scaling (MDS) analysis of the microbial restoration process was performed to visualize the variability of the microbial community, the impact of the freezing process, and the microbiota expansion itself. MDS plots showed a separation between the samples over time as a result of the microbial restoration; however, no significant differences were found between the restoration process using MOM or FMOM (Supplementary Figure 4).

To further identify confounding effects that may explain the variance observed among the samples, a PERMANOVA analysis was used. Interestingly, from all the different factors analyzed, only the storage of MOM (fresh or frozen) was not significant (*p* = 0.107) (Table 2). The main factor that explained the variability observed was the individual MOM (*R*^2^ = 0.066, *p* = 0.001), followed by race (*R*^2^ = 0.035), use of antibiotics at delivery (*R*^2^ = 0.034), infant’s gender (*R*^2^ = 0.032), and birth weight (*R*^2^ = 0.030).

**TABLE 2:** Contribution of the different factor to the diversity of the MOM microbiomes determined by PERMANOVA analysis.

**Factor**	***R*^2^**	***p*-value**
Maternal age	0.026	0.001
Gestational age	0.014	0.001
Birth weight	0.030	0.001
Milk type	0.026	0.001
Infant gender	0.032	0.001
Race	0.035	0.001
Delivery mode	0.016	0.001
Breastfeeding	0.014	0.001
Storage	0.005	0.107
Subject number	0.066	0.001
MEDAS	0.008	0.010
Antibiotics at delivery	0.034	0.001
Time points	0.012	0.080

In agreement with these observations, a PCoA and NMDS of the data regarding RM/MOM samples showed no clustering by MOM or FMOM samples over time; however, the clustering by infants that were breastfeeding directly reached significance after 4 h of incubation (Supplementary Figures 5A–D). PERMANOVA analysis showed that the highest contribution to the alpha diversity was accounted by the individual MOM at T4 (*R*^2^ = 0.5, *p* = 0.001) (Supplementary Figure 5E).

**FIGURE 3:**
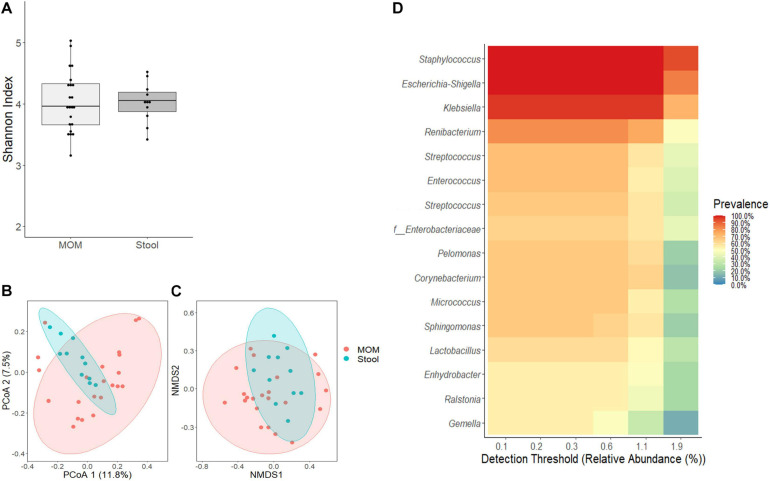
Comparative analyses of MOM (light gray box) and stool (gray box) microbiome. **(A)** Alpha diversity determined using Shannon index; statistical significance was determined using Kruskal–Wallis tests. **(B)** PCoA and **(C)** NMDS analysis of stool and MOM samples. PERMANOVA was used to analyze statistical significance regarding the distance between the samples. **(D)** Determination of core-taxa shared by stool and MOM samples. A minimum detection of 0.001 and a prevalence = 50/100 were used to define the core-taxa.

Next, we evaluated whether the confounding effects identified for RM would also have an impact on the infant’s intestinal microbiome. As shown in Figure 3A, no significant differences were observed between MOM and stools’ alpha diversity; however, significant differences were observed in their microbiome composition as evidenced by the clustering observed using PCoA (*R*^2^ = 0.0499, *p* = 0.004) and NMDS (*R*^2^ = 0.0589, *p* = 0.003) analysis (Figures 3B,C). A core-taxon constituted by 16 OTUs was identified between MOM and stools samples (Figure 3D), where the most prevalent genera (with 90–100% of prevalence) in the samples were *Staphylococcus*, *Escherichia*–*Shigella*, *Klebsiella*, and *Renibacterium*. Using PERMANOVA, we determined the main factors contributing to the variability of the microbiome were the sample type (*R*^2^ = 0.059, *p* = 0.001) (stool or milk), followed by infant’s gender (*R*^2^ = 0.049, *p* = 0.003) and race (*R*^2^ = 0.079, *p* = 0.011) (Table 3). The microbiota composition of the stool by themselves did not show significant differences regarding the different variables analyzed.

**TABLE 3:** Contribution of the different factors to the diversity of the microbiome in stool and MOM samples.

**Factor**	***R*^2^**	***p*-value**
Sample type	0.059	0.001
Maternal age	0.036	0.079
Birth weight	0.040	0.022
Infant gender	0.049	0.003
Race	0.079	0.011
Delivery mode	0.039	0.040
Subject number	0.026	0.597
Breastfeeding	0.033	0.123
Gestational age	0.029	0.387

### Freezing MOM Alters the Expansion Pattern of Few Genera in DHM

**FIGURE 4:**
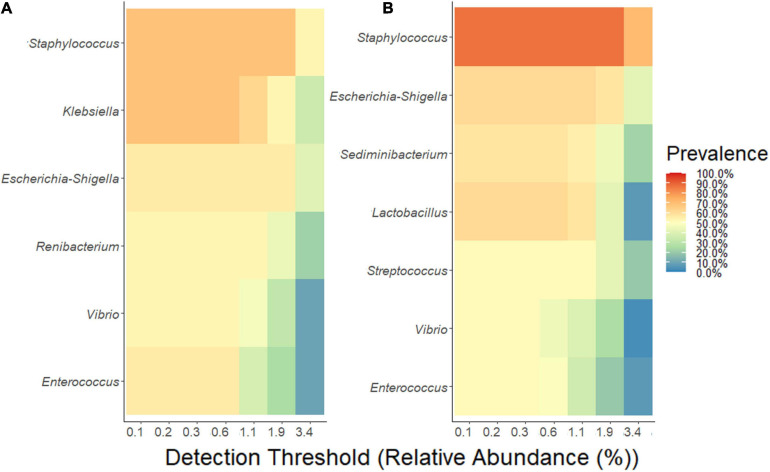
Determination of core-taxa shared by **(A)** MOM/RM and **(B)** FMOM/FRM samples after 4 h of incubation. A minimum detection of 0.001 and a prevalence = 50/100 were used to define the core-taxa.

The analyses of the microbial communities indicated that the relative abundance at phylum level is similar among the MOM and FMOM samples at T4 (Supplementary Figure 6). In order to determine if there was a core-taxon expanding differentially between MOM/FMOM and RM/FRM samples, the prevalence of the different OTUs was analyzed. As shown in Figure 4, a core-taxon that constituted seven different genera was identified in MOM-RM and FMOM-FRM. These core-taxa were very similar with a few variations in the OTUs forming the core. In MOM-RM samples (both RM10 and RM30), the most prevalent taxa were *Staphylococcus* and *Klebsiella*, found in 70% of the samples followed by *Escherichia-Shigella* (60%), *Enterococcus* (60%), *Renibacterium* (50%), and *Vibrio* (50%). FMOM-FRM core-taxa contained a high prevalence of *Staphylococcus* in 90% of the samples, followed by *Escherichia*–*Shigella* and *Lactobacillus* in 70% of the samples, whereas *Sediminibacterium* (60%), *Streptococcus* (50%), *Vibrio* (50%), and *Enterococcus* (50%) were found at lower frequency (Figure 4).

**FIGURE 5:**
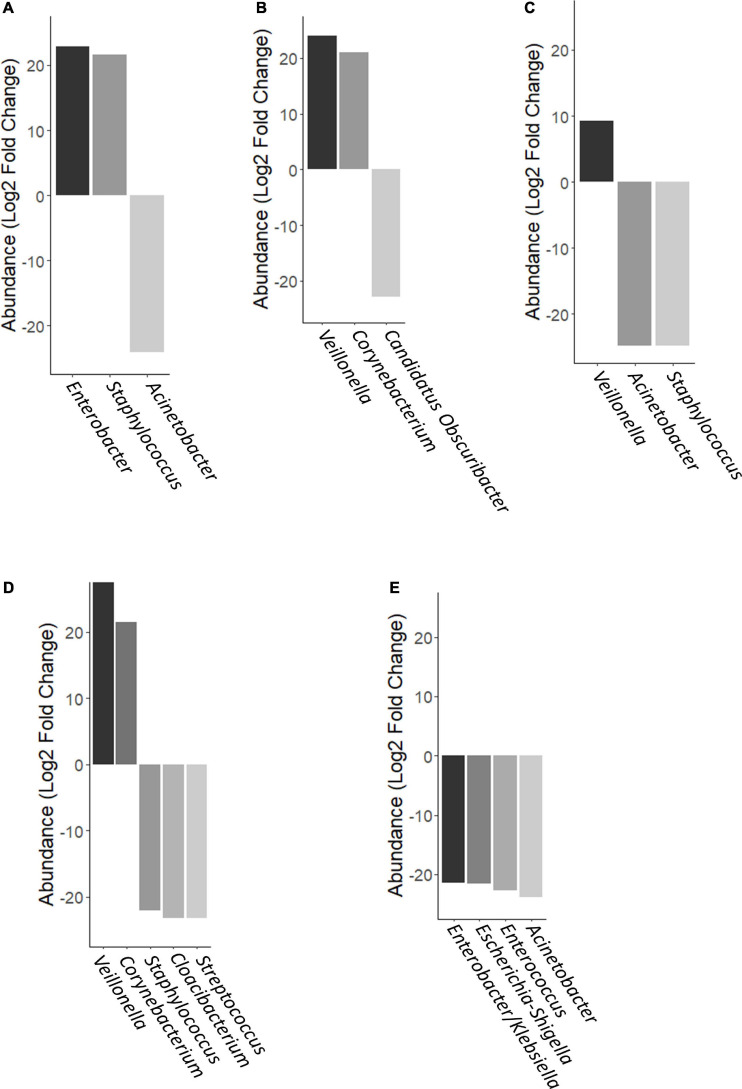
Differential abundance of OTUs using DESeq analysis. **(A)** FMOM compared to MOM at 4 h (T4), **(B)** MOM and RM30 at T4, **(C)** FMOM and FRM30 at T4, **(D)** MOM and RM10 at T4, and **(E)** FMOM and FRM10 at T4.

However, using negative binomial regression (DESeq2 analysis) for binary comparisons, we identified three OTUs where abundance was significantly different between MOM and FMOM after microbial expansion for 4 h (Figure 5A and Supplementary Table 1). Increased abundance of *Enterobacter* and *Staphylococcus* were found in FMOM compared with MOM at T4, whereas an increase of *Acinetobacter* was determined in MOM (*p* < 0.001) (Figure 5A and Supplementary Table 1). When MOM and RM30 samples were compared at T4, an increase of *Veillonella* and *Corynebacterium* was determined in MOM, whereas a significant increase in *Candidatus Obscuribacter* was observed in RM30 (*p* < 0.001) (Figure 5B and Supplementary Table 2). FMOM and FRM30 presented significant differences in the abundance of 3 OTUs, as well as an increase in *Veillonella* was determined in FMOM, whereas increases in *Acinetobacter* and *Staphylococcus* were observed in FRM30 at T4 (*p* < 0.001) (Figure 5C and Supplementary Table 3). DESeq2 analysis was performed to determine if any specific abundant OTUs were found to be fluctuating significantly between MOM/FMOM and RM10/FRM10 samples. When MOM and RM10 were compared, increases in *Veillonella* and *Corynebacterium* were observed in MOM samples at T4, whereas increases of *Staphylococcus*, *Cloacibacterium*, and *Streptococcus* were observed in RM10 samples (*p* < 0.001) (Figure 5D and Supplementary Table 4). Between the frozen samples, a different set of OTUs changed their abundance significantly between FMOM and FRM10 samples. Increases in *Enterobacter*/*Klebsiella*, *Escherichia-Shigella*, *Enterococcus*, and *Acinetobacter* were determined in FRM10 at T4 compared to FMOM (*p* < 0.001) (Figure 5E and Supplementary Table 5).

### Personalization of DHM With Fresh MOM or FMOM Results in Similar Stimulation of IL-8 Production in Caco-2 Cells

**FIGURE 6:**
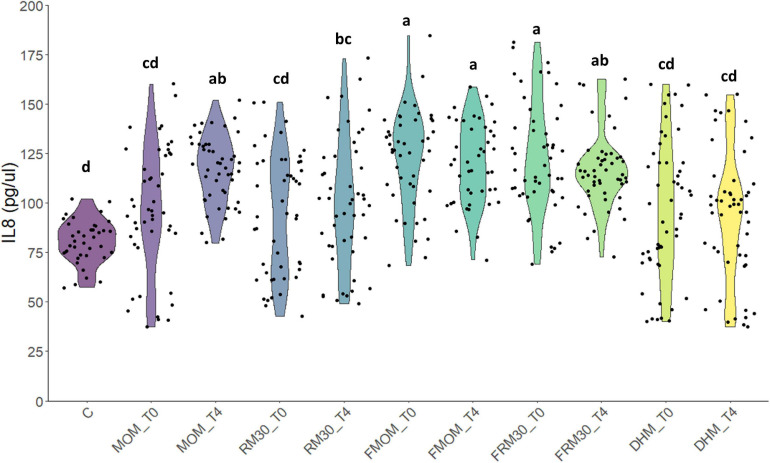
Effect of milk restoration on stimulation of IL-8 secretion in Caco-2 epithelial cells. ELISA quantification of IL-8 secretion in Caco-2 epithelial cells after 24 h of treatment with the different milk supernatants (MOM, RM30, FMOM, FRM30, and DHM) at time 0 (T0) and 4 h (T4). Untreated Caco-2 cells were used as control (C). The experiment was performed with biological and technical triplicates. ANOVA and Tukey honestly significant difference method were used to assign statistical significance for a *p* < 0.05.

Besides the macronutrients and micronutrients, MOM allows premature infants to incorporate immunomodulatory molecules such as immunoglobulins and cytokines helping to develop their immature immune system (Ballard and Morrow, 2013). Complementary to MOM, these components, the live microbiota in MOM, have an important role in early stimulation of the immune system. Next, it was investigated whether the personalization process as well as the storage of MOM at −20°C would modify the ability of MOM to stimulate the innate immune system in an intestinal epithelial cell line. To this end, defatted milk samples from MOM and FMOM as well as its derived RM samples were evaluated for IL-8 secretion in Caco-2 cells. Cells were treated with 4% of the milk samples and incubated for 24 h. After the incubation, the supernatants were collected, and IL-8 secretion was quantified. As small IL-8 is also present in MOM (Palkowetz et al., 1994; Kelleher and Lönnerdal, 2001), the concentration was determined and subtracted from the values obtained after incubation. It was found that the treatment of Caco-2 cells with 4% DHM did not have a significant difference when compared to untreated cells. The addition of MOM resulted in a significant increase in IL-8 secretion; however, microbial expansion for 4 h did not result in a significant further increase in IL-8 production. In contrast, FMOM resulted in significant IL-8 stimulation at T0 with no further changes after 4 h of incubation. Interestingly, FRM30 samples showed a significant production of IL-8 when compared to RM30 samples, DHM, and controls. In contrast, RM30 samples did not induce a significant stimulation of IL-8 at T0 or at T4 (Figure 6). These results are in agreement with the differential expansion of some microbial genera observed in the microbiome analyses. We hypothesize that the freezing step may allow for changes in the microbiota constitution in MOM that could be beneficial for expansion of bacteria with higher immune stimulatory potential, resulting in a similar level of IL-8 production as MOM.

## Discussion

The goal for this study was to determine if the freezing process has an impact on the live MOM’s microbiome composition and/or on their potential to restore the microbiota of DHM. Using culture-dependent techniques, we were able to determine similar loads of bacteria from MOM and FMOM samples in all the culture media used. The numbers of CFU/mL were in a range of 10^3^ to 10^7^, with similar counts of bacteria determined in a previous study (Marín et al., 2009; Cacho et al., 2017). Significant differences were found only in MOM/FMOM samples after 4 h of incubation in MRS, nutrient agar, and mannitol salt agar media with greater loads of bacteria in FMOM samples (Figures 1A–C). These observations are in agreement with a previous report where no differences in the load of bacteria between recently expressed MOM and FMOM had been observed even after 6 weeks of storage at −20°C (Marín et al., 2009). In addition, RM/FRM samples showed no statistically significant differences in the load of bacteria regardless if the microbial expansion was made using recently expressed MOM or FMOM. These results suggest that the freezing storage of MOM does not affect the intrinsic bacterial load in fresh MOM samples. This observation supports the NICU’s strategy of freezing small amounts of recently expressed MOM without losing any of MOM’s properties. In addition, the results presented here support the use of RM or FRM to provide infants a better nutritional option rather than formula or fortified DHM.

In addition, similar alpha diversity of the microbiomes was observed when MOM/FMOM and RM/FRM were compared. These observations highlight an important attribute of the personalization process, not only could MOM microbiota be restored in DHM, but also the diversity is not modified over time. These results are significant as a decrease in diversity is related to the differential expansion of a few microbial species, which may result in an unbalanced RM. As indicated earlier, many genetic and environmental variables contribute to the microbial diversity in MOM. When different factors contributing to the variability of the microbiota such as maternal age, gestational age, weight of birth, infants’ gender, race, mode of delivery, MEDAS, and others were analyzed, the freezing storage of MOM was the only factor that did not show significant effects in the diversity of the samples. As expected, the overall abundance of the different phylum identified in MOM showed great similarity between MOM and FMOM samples. Although our mothers had a similar non-adherence to an MD, our study was the first to incorporate assessment of a diet related to the MOM microbiome in humans and mothers who delivered a preterm infant in the United States. However, a detailed nutritional assessment of the mother should be performed in order to thoroughly investigate the impact of the diet in the diversity of the human milk microbiota.

Enteral nutrition is not only the most important source of nutrients but also a main factor in the development of the infant’s gastrointestinal microbiome and its immune maturation (Stewart et al., 2018; Vatanen et al., 2018). The human milk microbiome has been reported to be modified by antibiotic administration, mastitis, obesity, mode of delivery, and even social patterns (environment, behavior, and social networks) (Cabrera-Rubio et al., 2012; Jiménez et al., 2015; Meehan et al., 2018; Hermansson et al., 2019; Moossavi et al., 2019). While a lot of progress has been done to understand how MOM microbiome is shaped, the large number of factors involved in its composition hinders the progress toward the design of a synthetic community that may re-establish the microbiome in DHM. Therefore, RM could be a valuable strategy to provide infants their own personalized DHM maximizing the chances of the early establishment of MOM-driven beneficial microbiota. Indeed, lower incidence and risk of disease have been reported in MOM-fed compared to formula-fed infants (Geddes and Prescott, 2013; Oddy, 2017). To evaluate the potential impact of RM milk on immune stimulation, the production of IL-8 upon addition of RM milk was assessed and compared to MOM and DHM. It was found that the addition of RM30 did not exacerbate MOM’s own effect. Interestingly, when the IL-8 secretion levels were compared between MOM/RM and FMOM/FRM samples, slightly greater levels of IL-8 were observed in the Caco-2 cells treated with FMOM/FRM samples at T4. These results suggest that not only are the microbes conserved, but also the metabolites/compounds present in MOM could similarly be replicated in RM. We have recently reported that MOM and RM30 metabolomic profiles differ significantly from DHM (Torrez Lamberti et al., 2020). DHM is pasteurized because of safety concerns to reduce the presence of potential pathogens; however, the pasteurization process not only destroys the microbiota but also inactivates a great amount of valuable compounds as well (Evans et al., 1978; Landers and Updegrove, 2010; Montjaux-Régis et al., 2011; García-Lara et al., 2013; Marx et al., 2014). Therefore, personalization of DHM with FMOM could be a suitable strategy to provide enteral feeding to premature infants and provide all the intrinsic properties of MOM that may be lost if MOM is not stored at −20°C.

In summary, FMOM showed great similarities with MOM, allowing equivalent inoculum usage for personalization of DHM and restoring the unique and intrinsic microbiota present in each mother. This work establishes the basis for a suitable strategy providing personalized milk for the premature infant when limited quantities of MOM are available for enteral feeding. This strategy supports optimal nutritional feeding and immune protection while avoiding formula-feeding risks. However, further studies are needed to fully assess the effects of RM *in vivo*.

## Data Availability Statement

The original contributions presented in the study are included in the article/Supplementary Material, further inquiries can be directed to the corresponding author/s.

## Ethics Statement

The studies involving human participants were reviewed and approved by the University of Florida’s Institutional Review Board. The patients/participants provided their written informed consent to participate in this study.

## Author Contributions

NH, MB, JN, LP, and GL conceptualized the study. MT, NH, MB, ED-S, ST, LP, and GL contributed to the sample processing and data analyses. MT, NH, MB, JN, GL, and LP contributed to the manuscript writing (review and editing). All authors have read and agreed to the published version of the manuscript.

## Conflict of Interest

The authors declare that the research was conducted in the absence of any commercial or financial relationships that could be construed as a potential conflict of interest.
