# Supplementary figures and images for: Frozen Mother’s Own Milk Can Be Used Effectively to Personalize Donor Human Milk
Source: Front Microbiol. 2021 Apr 14;12:656889. doi: 10.3389/fmicb.2021.656889 (PMC8079756; doi:10.3389/fmicb.2021.656889)

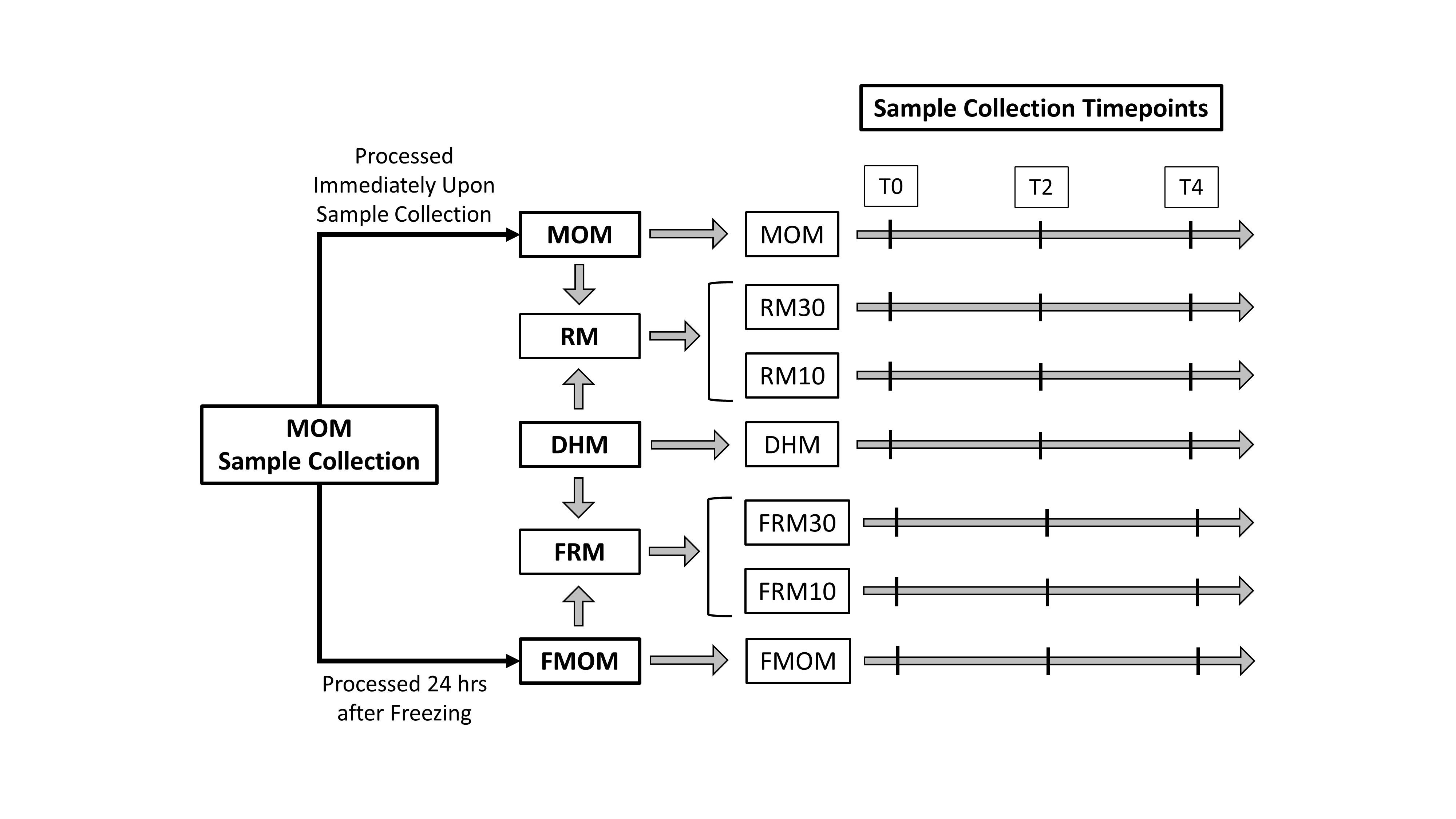

Supplement: Supplementary file 1 [file Image_1.TIF]

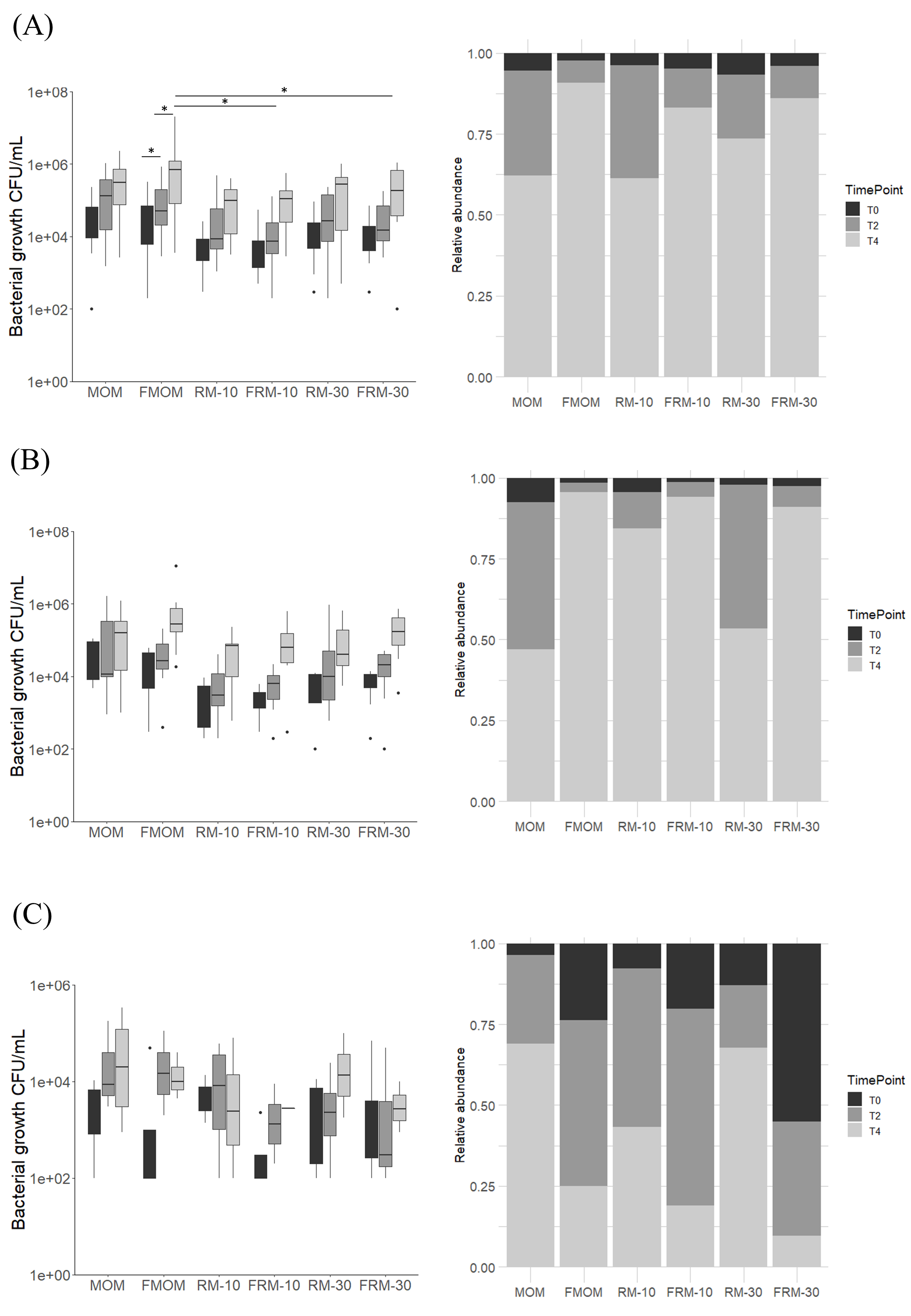

Supplement: Supplementary file 2 [file Image_2.TIF]

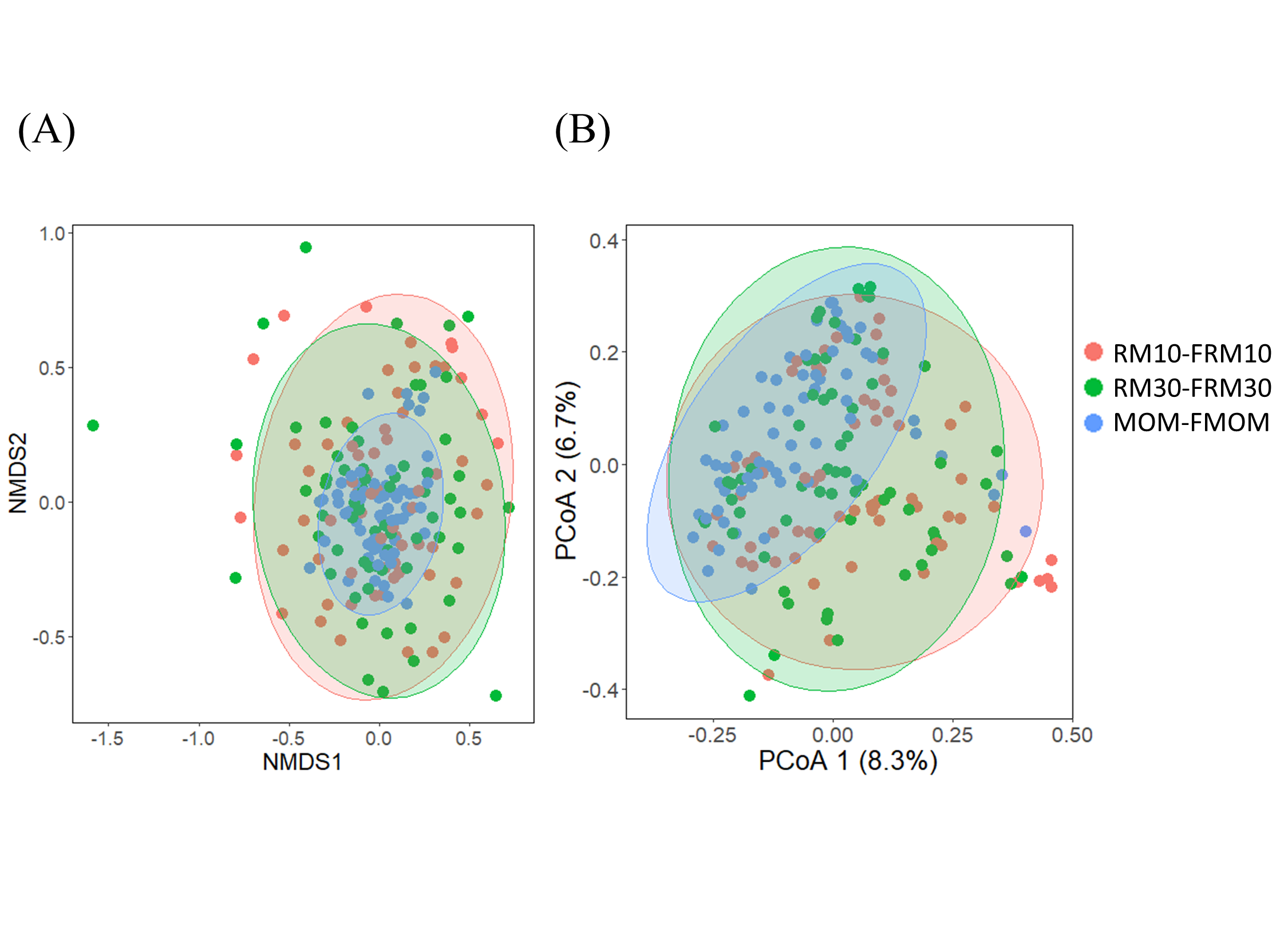

Supplement: Supplementary file 3 [file Image_3.TIF]

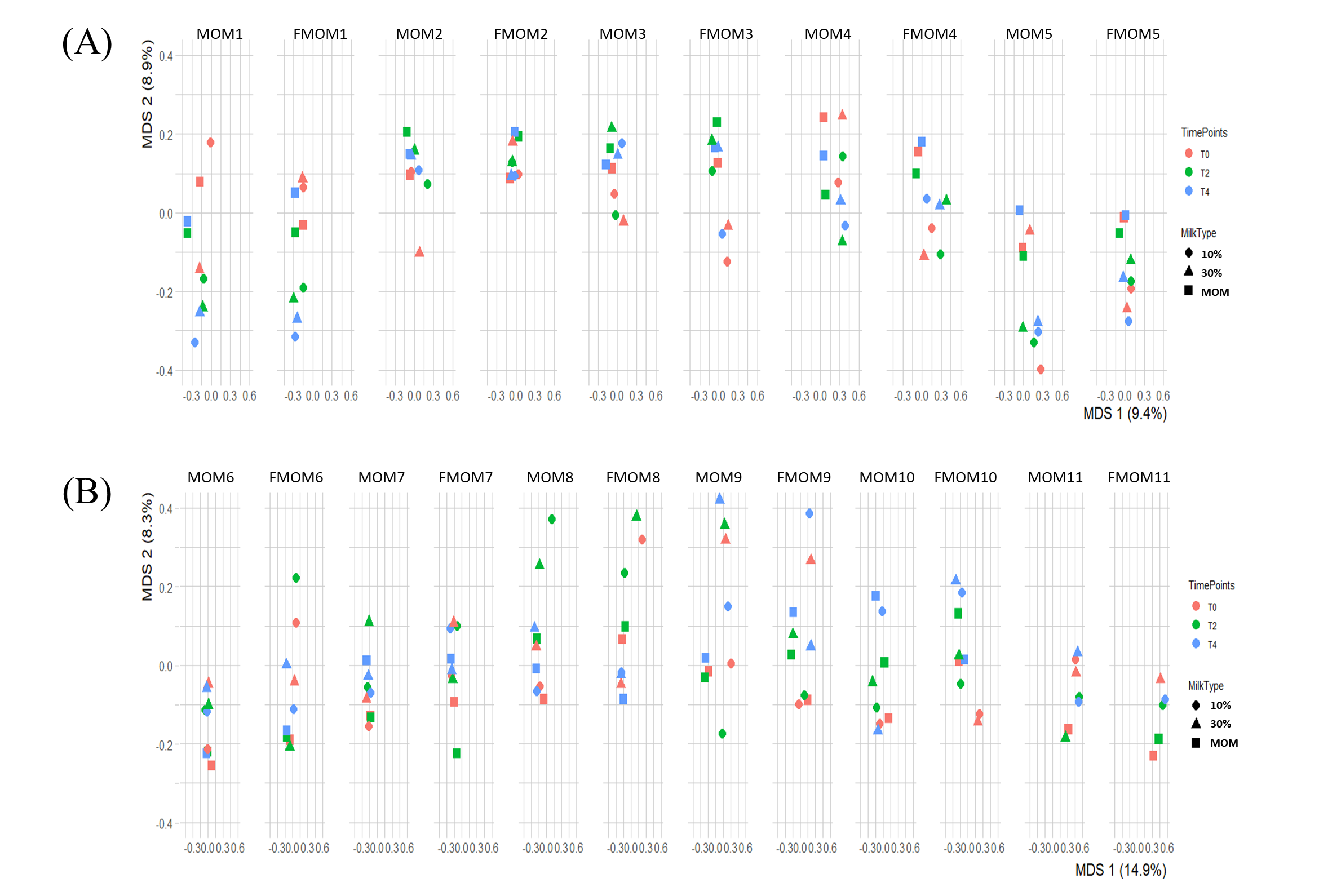

Supplement: Supplementary file 4 [file Image_4.TIF]

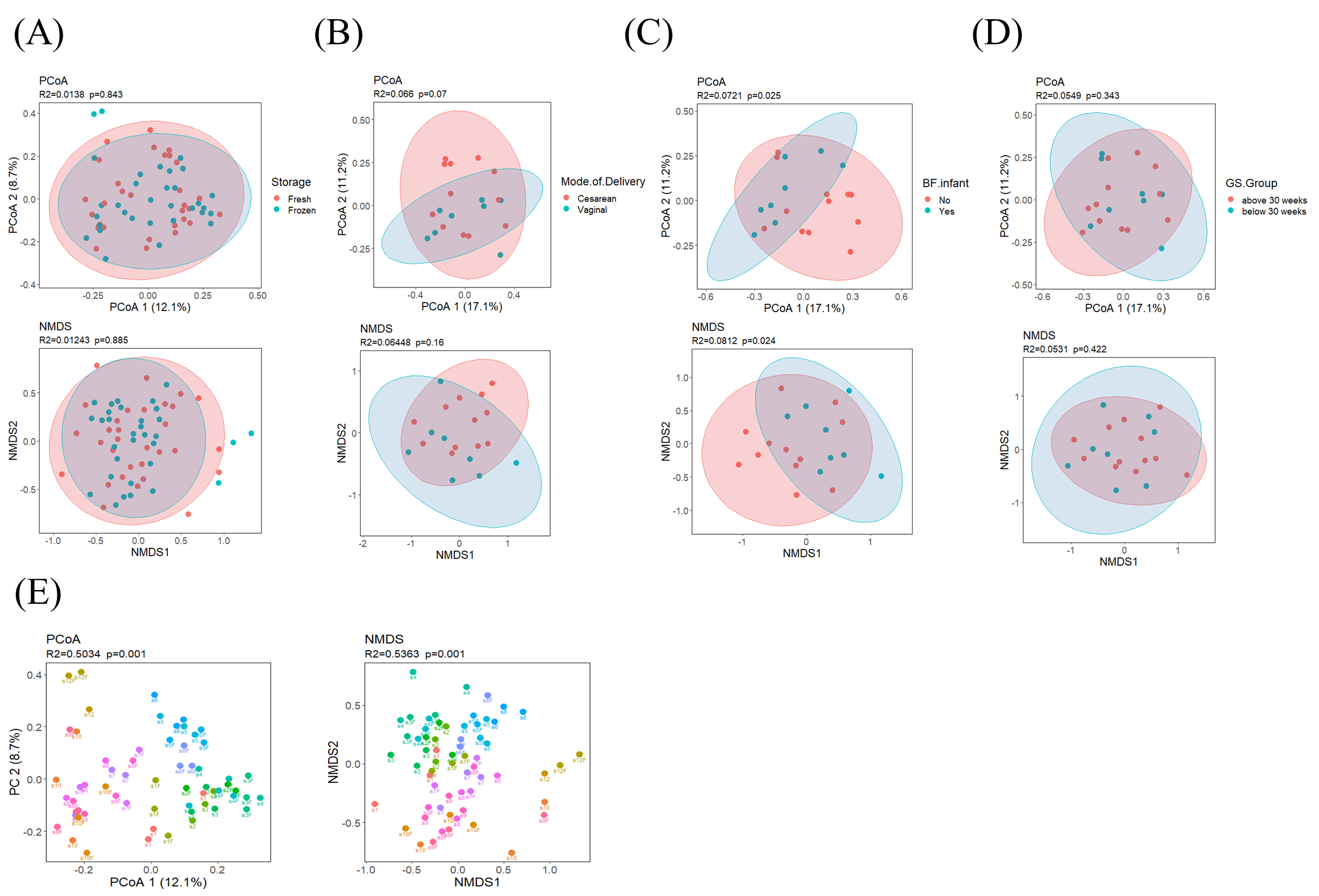

Supplement: Supplementary file 5 [file Image_5.TIF]

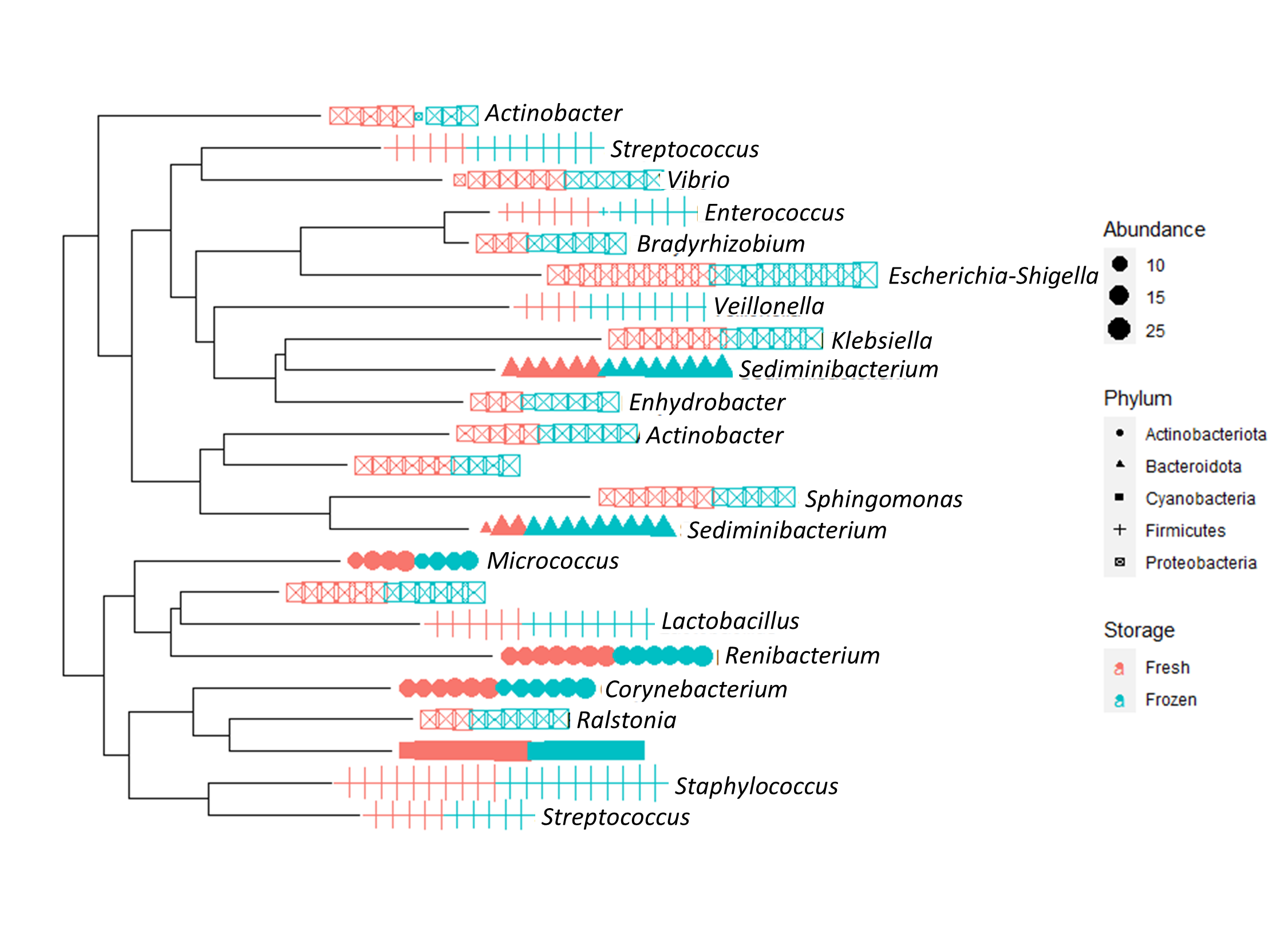

Supplement: Supplementary file 6 [file Image_6.TIF]
