# Supplementary material for: Frozen Mother’s Own Milk Can Be Used Effectively to Personalize Donor Human Milk
Source: Front Microbiol. 2021 Apr 14;12:656889. doi: 10.3389/fmicb.2021.656889 (PMC8079756; doi:10.3389/fmicb.2021.656889)
Supplement: Supplementary file 8 [file Table_1.docx]

**Supplementary Table 1.** OTU differential abundance between MOM and FMOM samples at T4.

| Genus | Family | Phylum | log2FoldChange | p-adj |
| --- | --- | --- | --- | --- |
| Acinetobacter | *Moraxellaceae* | *Proteobacteria* | -24.11 | 4.28E-14 |
| Staphylococcus | *Staphylococcaceae* | *Firmicutes* | 21.65 | 1.05E-11 |
| Enterobacter | *Enterobacteriaceae* | *Proteobacteria* | 22.88 | 6.16E-13 |
