# Supplementary material for: Frozen Mother’s Own Milk Can Be Used Effectively to Personalize Donor Human Milk
Source: Front Microbiol. 2021 Apr 14;12:656889. doi: 10.3389/fmicb.2021.656889 (PMC8079756; doi:10.3389/fmicb.2021.656889)
Supplement: Supplementary file 9 [file Table_2.docx]

**Supplementary Table 2.** OTU differential abundance between MOM and RM30 samples at T4.

| Genus | Family | Phylum | log2FoldChange | p-adj |
| --- | --- | --- | --- | --- |
| Candidatus Obscuribacter | *Obscuribacteraceae* | *Cyanobacteria* | -22.89 | 1.10E-14 |
| Corynebacterium | *Corynebacteriaceae* | *Actinobacteriota* | 21.07 | 1.16E-12 |
| Veillonella | *Veillonellaceae* | *Firmicutes* | 23.98 | 4.87E-16 |
