# Supplementary material for: Frozen Mother’s Own Milk Can Be Used Effectively to Personalize Donor Human Milk
Source: Front Microbiol. 2021 Apr 14;12:656889. doi: 10.3389/fmicb.2021.656889 (PMC8079756; doi:10.3389/fmicb.2021.656889)
Supplement: Supplementary file 10 [file Table_3.docx]

**Supplementary Table 3.** OTU differential abundance between FMOM and FRM30 samples at T4.

| Genus | Family | Phylum | log2FoldChange | p-adj |
| --- | --- | --- | --- | --- |
| Staphylococcus | *Staphylococcaceae* | *Firmicutes* | -24.79 | 3.85E-15 |
| Veillonella | *Veillonellaceae* | *Firmicutes* | 9.23 | 3.29E-05 |
| Acinetobacter | *Moraxellaceae* | *Proteobacteria* | -24.78 | 3.85E-15 |
