# Supplementary material for: Frozen Mother’s Own Milk Can Be Used Effectively to Personalize Donor Human Milk
Source: Front Microbiol. 2021 Apr 14;12:656889. doi: 10.3389/fmicb.2021.656889 (PMC8079756; doi:10.3389/fmicb.2021.656889)
Supplement: Supplementary file 11 [file Table_4.docx]

**Supplementary Table 4.** OTU differential abundance between MOM and RM10 samples at T4.

| Genus | Family | Phylum | log2foldchange | p-adj |
| --- | --- | --- | --- | --- |
| Streptococcus | *Streptococcaceae* | *Firmicutes* | -23.24 | 2.13E-13 |
| Staphylococcus | *Staphylococcaceae* | *Firmicutes* | -22.12 | 2.82E-12 |
| Veillonella | *Veillonellaceae* | *Firmicutes* | 23.13 | 2.13E-13 |
| Corynebacterium | *Corynebacteriaceae* | *Actinobacteriota* | 21.50 | 1.12E-11 |
| Cloacibacterium | *Weeksellaceae* | *Bacteroidota* | -23.22 | 2.13E-13 |
| Veillonella | *Veillonellaceae* | *Firmicutes* | 24.41 | 2.43E-14 |
