# Supplementary material for: Frozen Mother’s Own Milk Can Be Used Effectively to Personalize Donor Human Milk
Source: Front Microbiol. 2021 Apr 14;12:656889. doi: 10.3389/fmicb.2021.656889 (PMC8079756; doi:10.3389/fmicb.2021.656889)
Supplement: Supplementary file 12 [file Table_5.docx]

**Supplementary Table 5.** OTU differential abundance between FMOM and FRM10 samples at T4.

| Genus | Family | Phylum | log2FoldChange | p-adj |
| --- | --- | --- | --- | --- |
| Enterobacter/Klebsiella | *Enterobacteriaceae* | *Proteobacteria* | -21.43 | 2.45E-11 |
| Enterococcus | *Enterococcaceae* | *Firmicutes* | -22.73 | 1.66E-12 |
| Acinetobacter | *Moraxellaceae* | *Proteobacteria* | -23.89 | 1.40E-13 |
| Escherichia-Shigella | *Enterobacteriaceae* | *Proteobacteria* | -21.55 | 2.39E-11 |
